# Chronic Suppurative Otitis Media Patient Presenting With Hyperhomocysteinemia in Granulomatosis With Polyangiitis

**DOI:** 10.7759/cureus.38412

**Published:** 2023-05-01

**Authors:** Dharam P Bansal, Ram K Jat, Medha Gupta

**Affiliations:** 1 General Medicine, Mahatma Gandhi Medical College and Hospital, Jaipur, IND

**Keywords:** mthfr, c-anca, cyclophosphamide, systemic vasculitis, hyperhomocysteinemia, cerebral venous sinus thrombosis, hypertrophic pachymeningitis, wegner’s granulomatosis, granulomatosis with polyangiitis

## Abstract

Granulomatosis with Polyangiitis (GPA) can present with Cerebral Venous Sinus Thrombosis (CVST), Chronic Suppurative Otitis Media, and Lower Motor Neuron (LMN) Facial Palsy. However, an association between CVST and Hyperhomocysteinemia in GPA has not previously been reported. Here, we report a case of CVST and Hyperhomocysteinemia in Proteinase 3 anti-neutrophil cytoplasmic antibody (PR3-ANCA) positive GPA without renal involvement.

## Introduction

Granulomatosis with Polyangiitis (GPA), formerly known as Wegner's granulomatosis, is characterized by necrotizing granulomatous inflammation, usually involving the upper and lower respiratory tracts with nodules, alveolar hemorrhage, and necrotizing glomerulonephritis. However, any organ system could be affected during disease progression [1-3]. Renal involvement is the most common at 18-77 %, Central Nervous System (CNS) involvement was 1-8 %, and Otitis Media was up to 25-44% % in GPA patients [4]. A recent case report has revealed an association between Cerebral Venous Sinus Thrombosis (CVST) and Hypertrophic Pachymeningitis (HP) in patients with Proteinase 3 anti-neutrophil cytoplasmic antibody (PR3-ANCA) positive GPA [5]. There is a clear relationship between CVST and Hyperhomocysteinemia [6]. A few studies have reported deep venous thrombosis (DVT) as venous involvement in GPA [7].

## Case presentation

**Figure 1:**
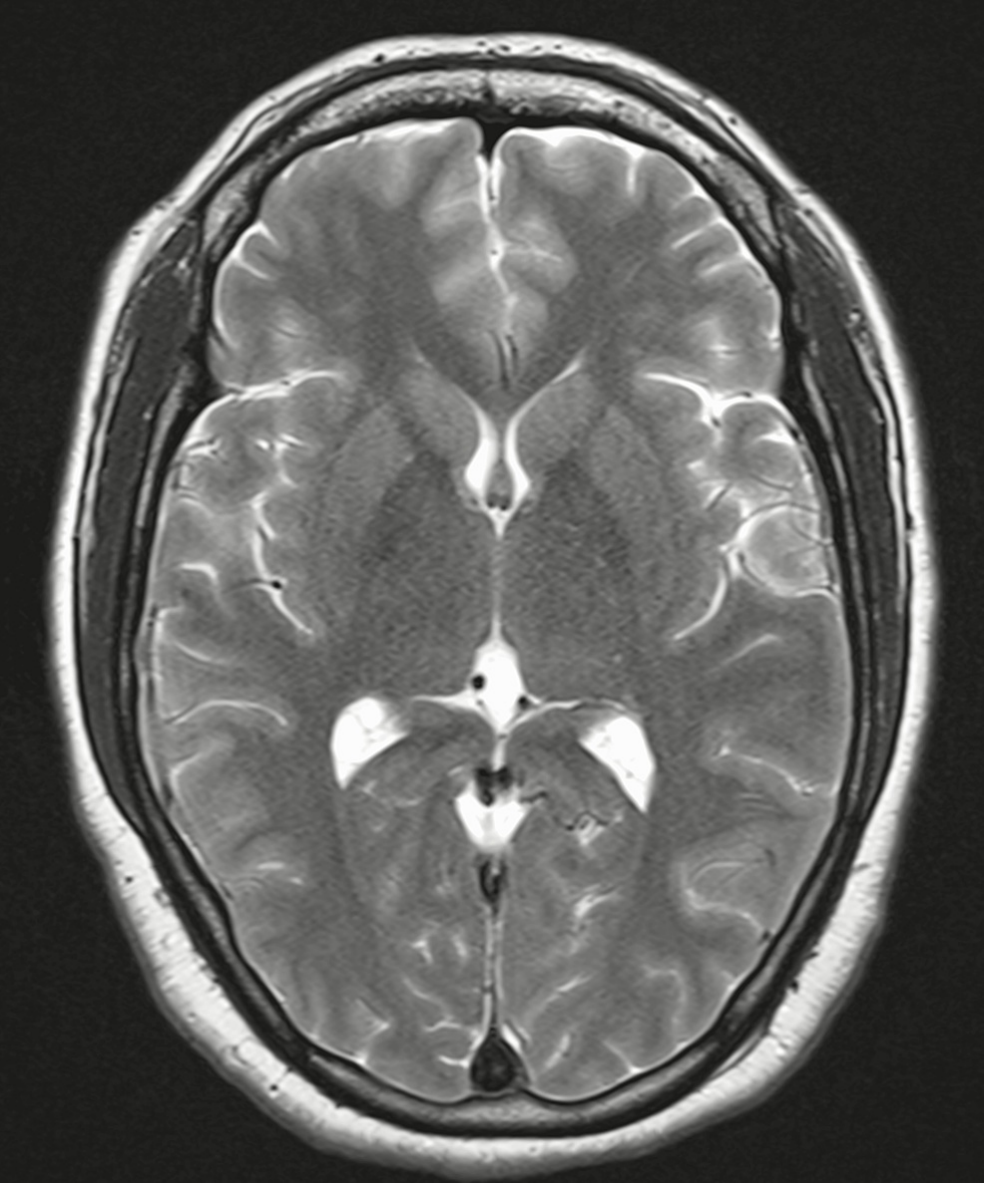
Contrast Enhanced MRI Brain - Cerebral Venous Sinus Thrombosis A non-invasive diagnostic procedure that uses a combination of a large magnet, radio frequencies, and a computer to produce detailed images of organs and structures within the body without the use of damaging ionizing radiation. There are filling defects within the superior sagittal sinus, left transverse & sigmoid sinuses, and the left jugular bulb. Filling defects are also seen in the right transverse and straight sinus and the Galen region's vein. Cortical veins draining into these sinuses appear engorged, suggesting thrombosis.

**Figure 2:**
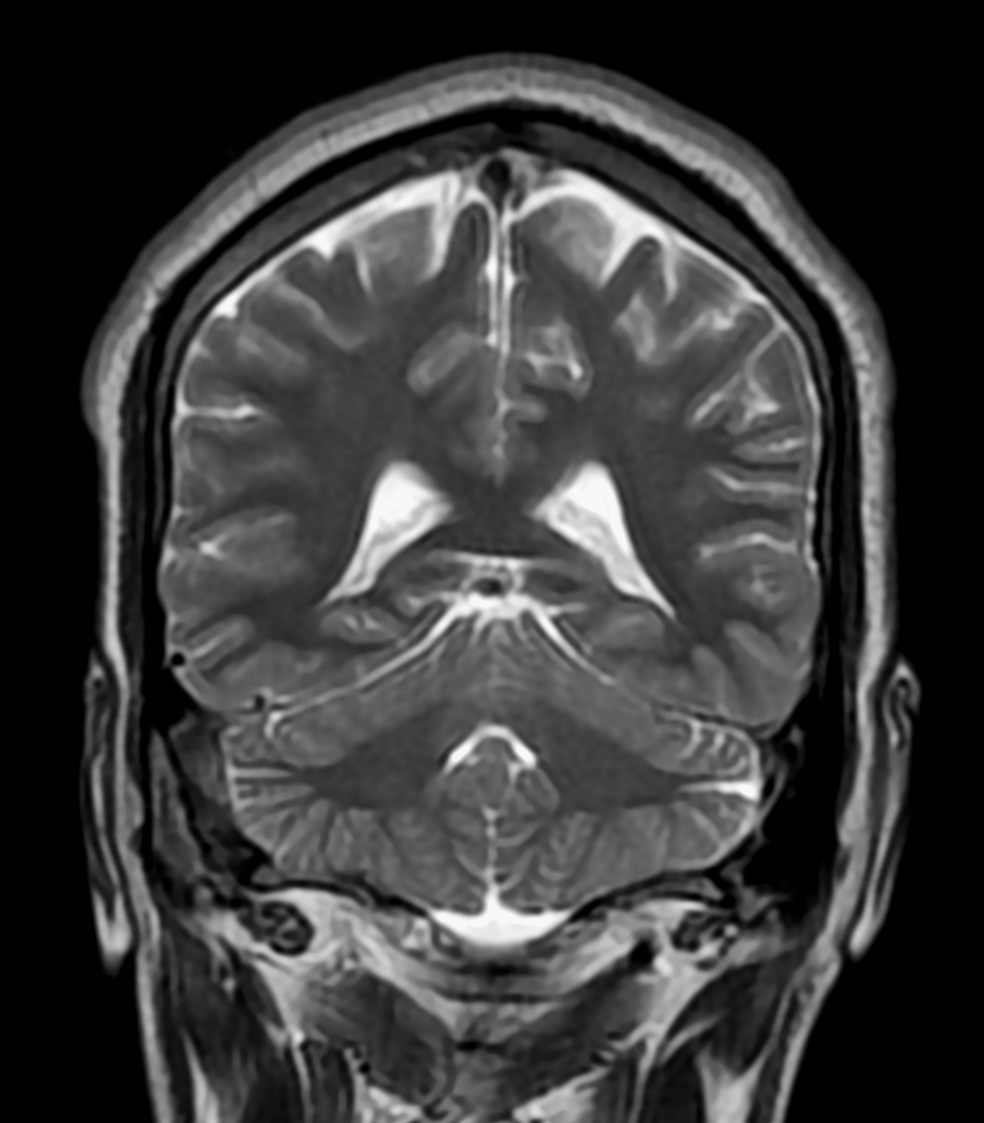
Contrast Enhanced MRI Brain - Cerebral Venous Sinus Thrombosis A non-invasive diagnostic procedure that uses a combination of a large magnet, radio frequencies, and a computer to produce detailed images of organs and structures within the body without the use of damaging ionizing radiation. There are filling defects within the superior sagittal sinus, left transverse & sigmoid sinuses, and the left jugular bulb. Filling defects are also seen in the right transverse and straight sinus and the Galen region's vein. Cortical veins draining into these sinuses appear engorged, suggesting thrombosis.

**Figure 3:**
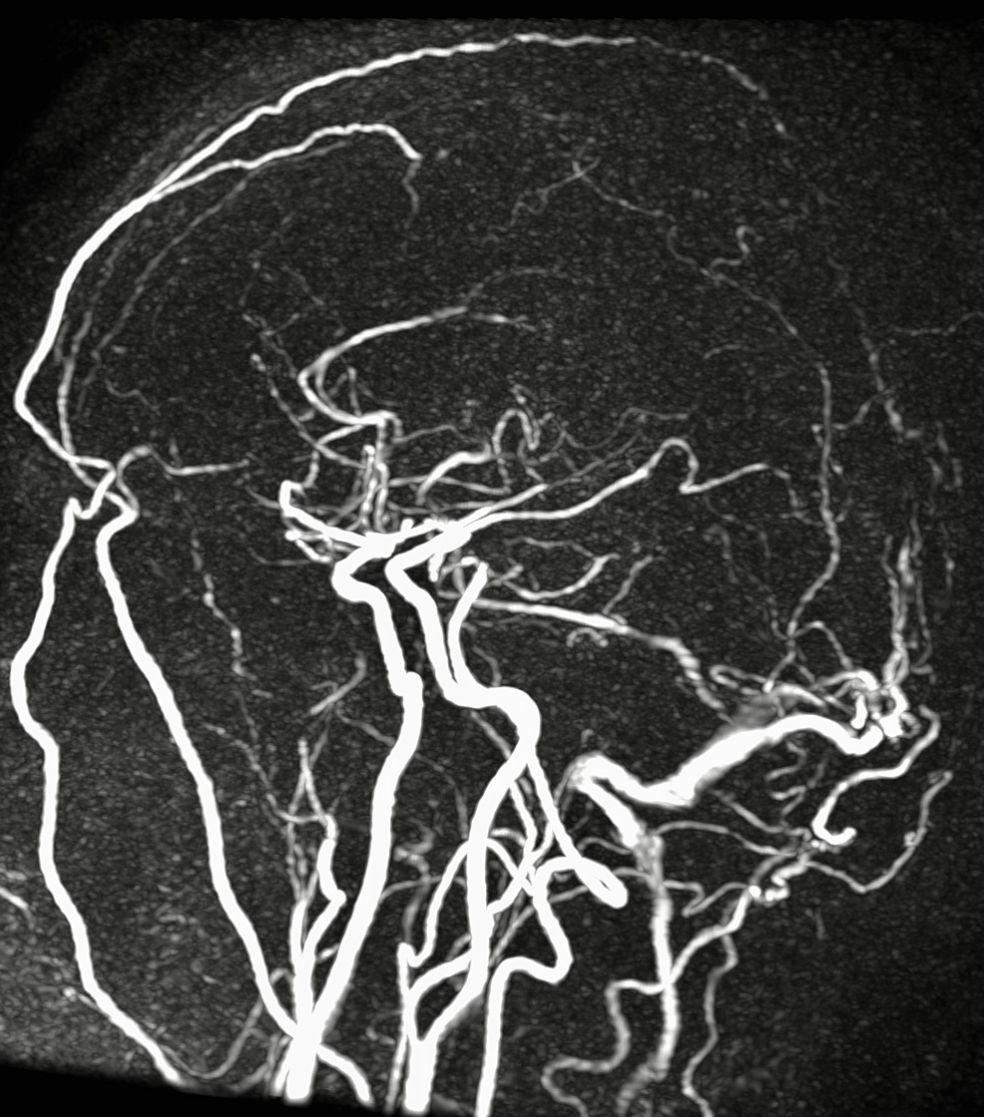
MRI Brain Venogram - Cerebral Venous Sinus Thrombosis A non-invasive diagnostic procedure that uses a combination of a large magnet, radio frequencies, and a computer to produce detailed images of organs and structures within the body without the use of damaging ionizing radiation. The mid and posterior parts of the superior sagittal sinus show no flow in venography. There is non-visualization of flow in the right transverse, sigmoid sinus, and jugular bulb. The proximal aspect of the left transverse sinus also shows partial loss of flow. No obvious flow was seen in Galen's straight sinus and vein- these findings suggest cerebral venous sinus thrombosis.

A 34-year-old male presented to General Medicine Outpatient Department with c/o low-grade fever without chills for five months, gradually culminating in persistent severe dry cough in the last three months and started having blood in sputum in the last two weeks. He took antibiotics and antipyretics, but there was no relief. He had a history of hospitalization two years ago for severe headaches and a single seizure episode. He was diagnosed with Cerebral Venous Sinus Thrombosis on MRI Brain Venogram (Figure 1-3), and serum Homocysteine was 234.9 µmol/L (4.7 - 14.8). After taking Acenocoumarol 2 mg every alternate day, he remained asymptomatic and stopped taking it after four weeks as he felt he was completely alright.

**Figure 4:**
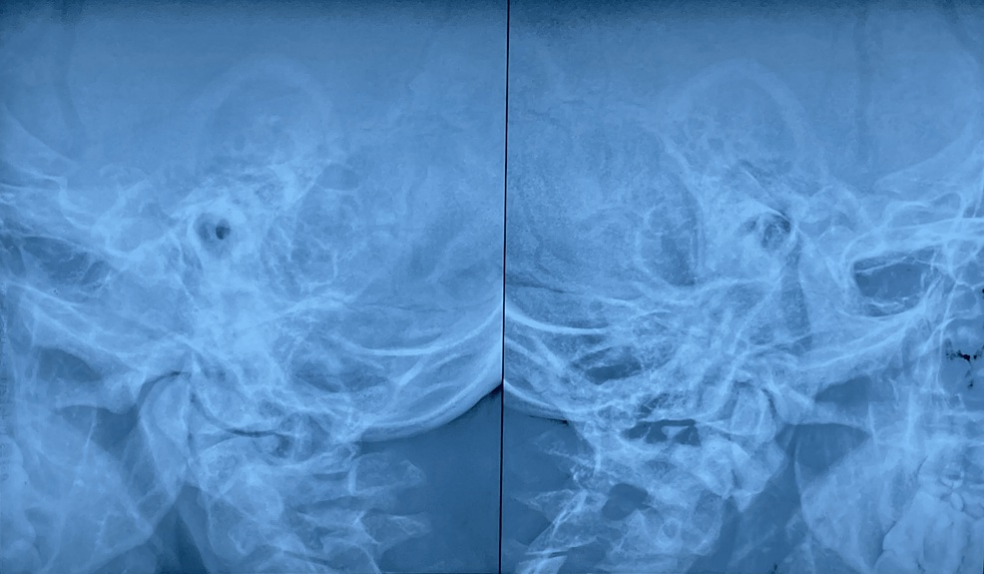
X-ray Mastoid X-ray - X-rays are a form of ionizing radiation, it’s an imaging study that takes pictures of bones and soft tissues. Air Fluid levels are destroyed & Mastoid air cells are obliterated.

Three months ago, he complained of pain in his right ear with hearing loss and drooping of the right angle of his mouth. He was treated for Right Chronic Suppurative Otitis Media and Lower Motor Neuron Facial Palsy (Figure 4) with Antibiotics and Acyclovir for three weeks. Now he presented with difficulty breathing; on examination, there was pallor, right infra-scapular crepitations with bronchial breath sounds, and drooping of the right side of the mouth with the inability to close the right eye completely.

**Figure 5:**
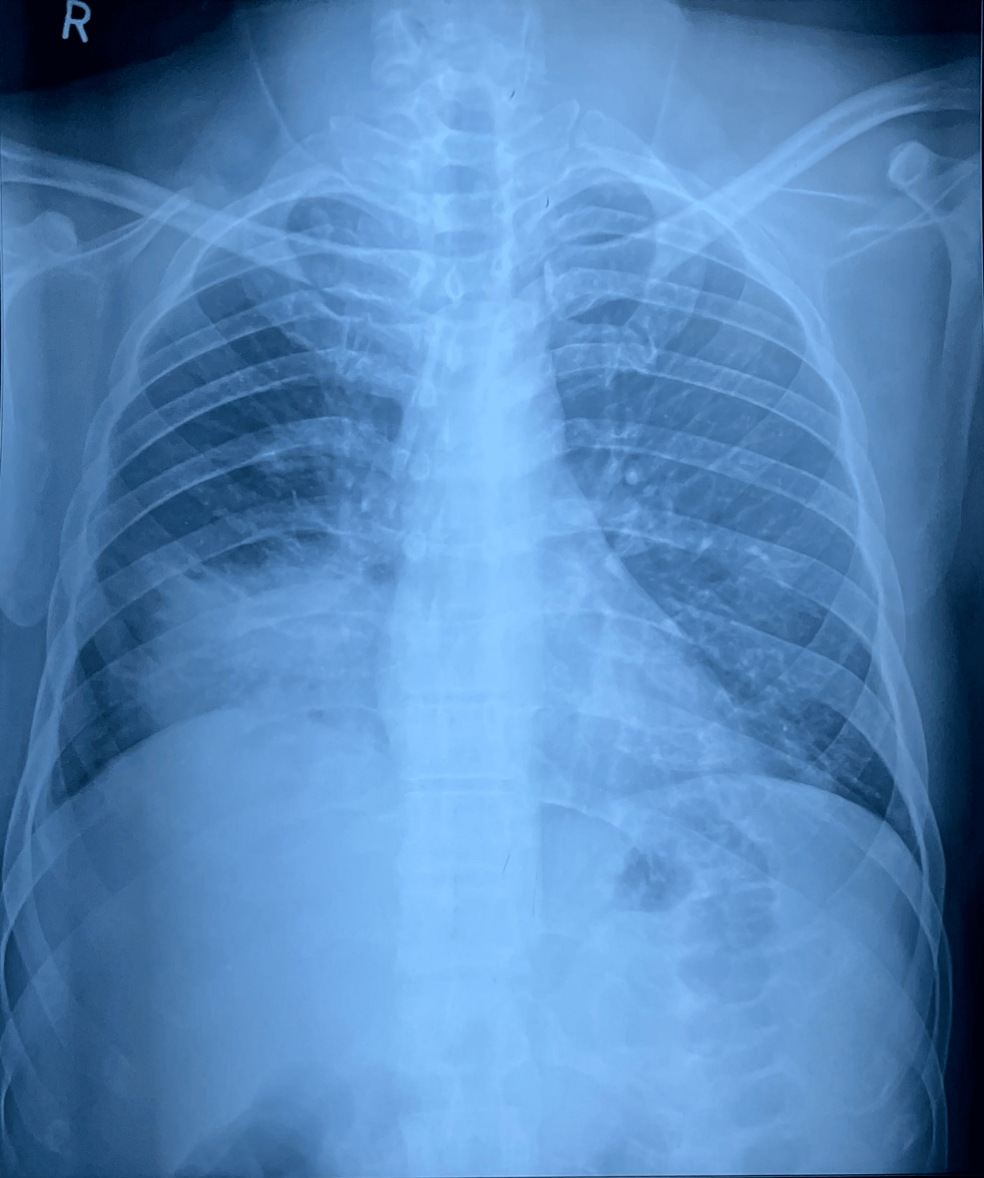
Chest X-ray Day 1 X-ray - X-rays are a form of ionizing radiation, an imaging study that takes pictures of bones and soft tissues. Lower zone homogenous consolidation, the encroaching periphery of the lung.

**Figure 6:**
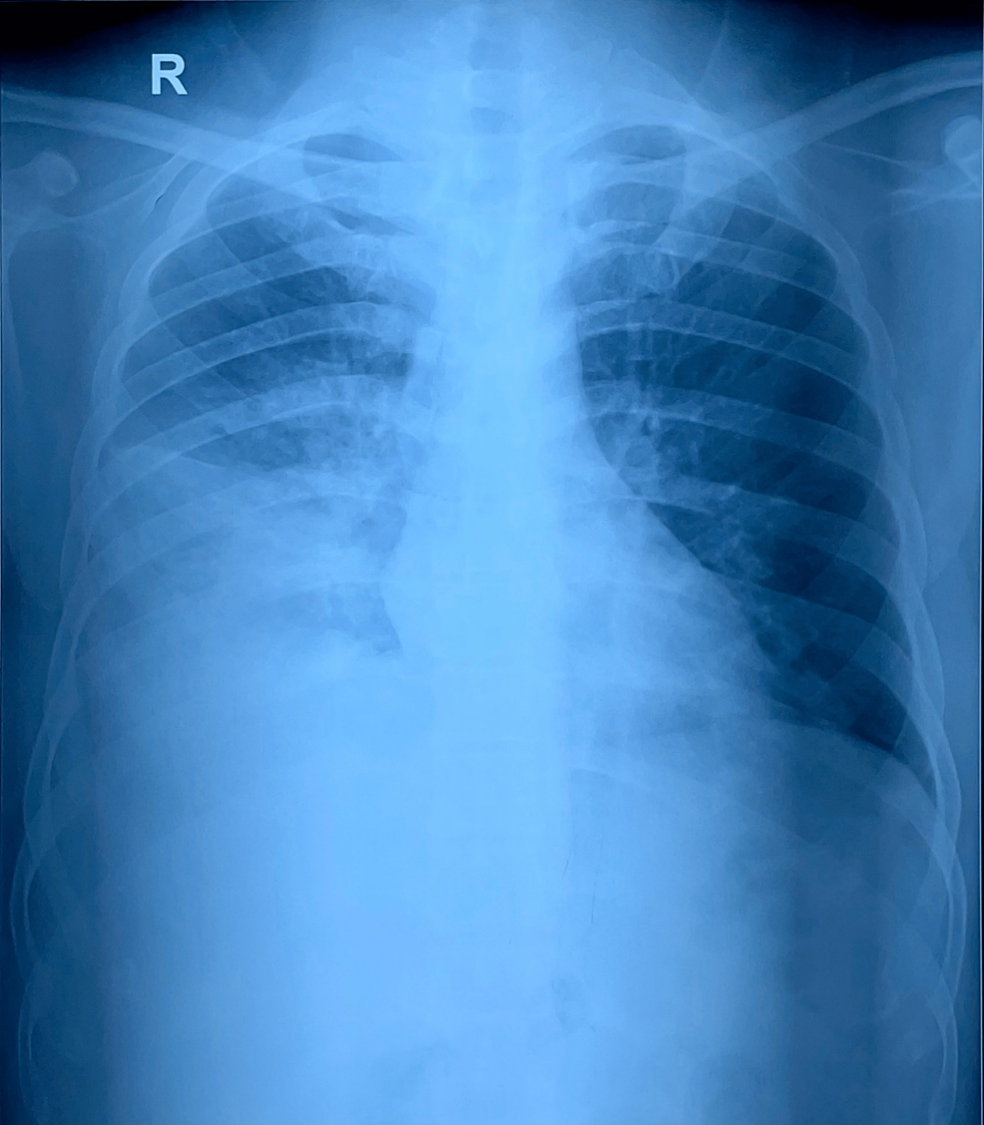
Chest X-ray Day 3 X-ray - X-rays are a form of ionizing radiation, an imaging study that takes pictures of bones and soft tissues. Lower zone homogenous consolidation covering the periphery of the lung.

**Figure 7:**
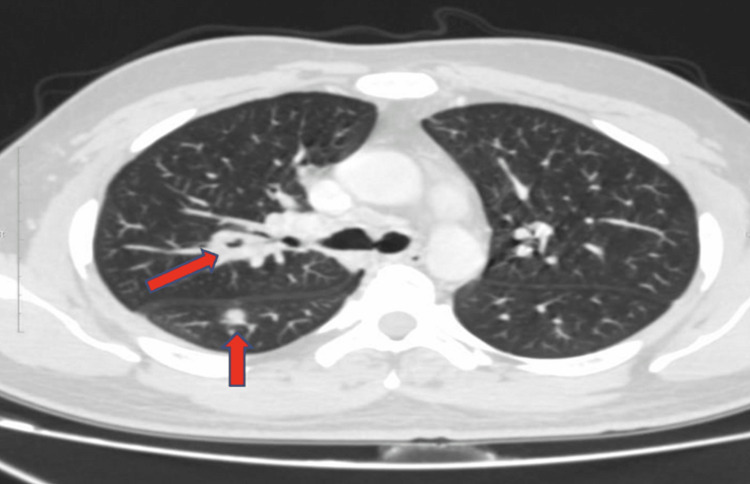
CECT Thorax CECT - Contrast Enhanced Computerized Tomography It's a diagnostic imaging tool to create detailed images of internal organs, bones, soft tissue, and blood vessels. Intravenous contrast dye is ingested into the body, which helps provide a detailed view of the blood vessels. Multiple noncavitary and cavitary nodules are seen in bilateral lung fields, predominantly in bilateral mid and lower lobes. Mild right pleural effusion is seen with subsegmental basal atelectatic changes. Sub-centimeter-sized lymph nodes are seen in the mediastinum and hilum.

**Figure 8:**
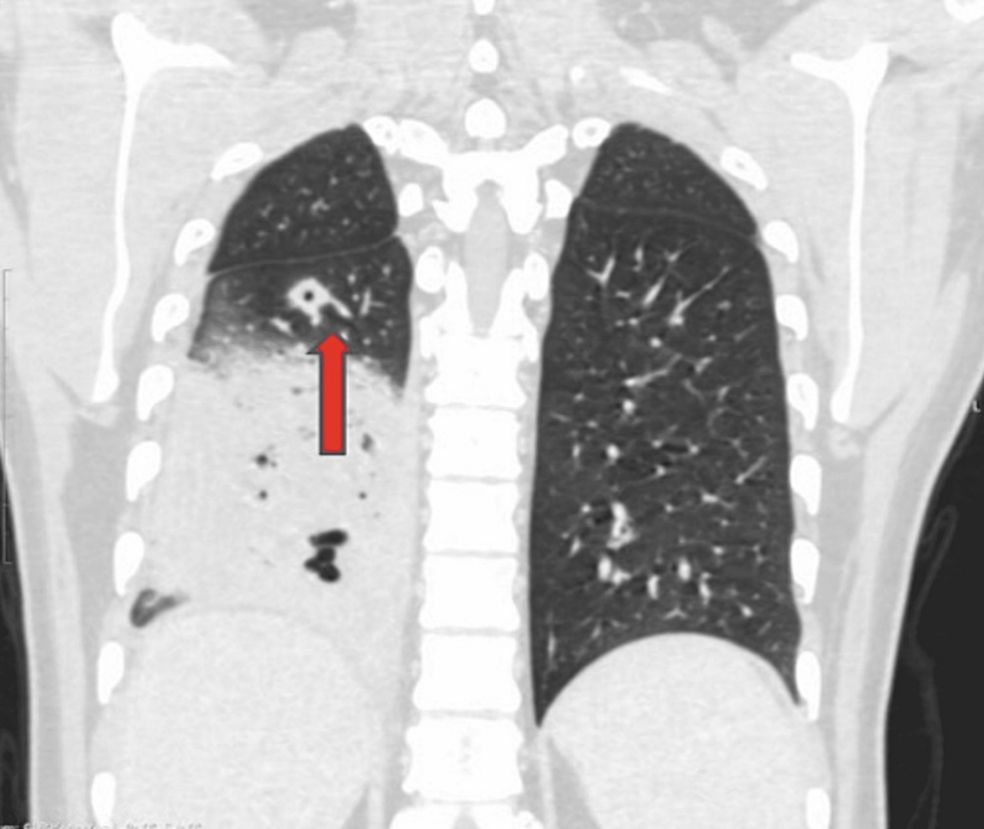
CECT Thorax CECT - Contrast Enhanced Computerized Tomography It's a diagnostic imaging tool to create detailed images of internal organs, bones, soft tissue, and blood vessels. Intravenous contrast dye is ingested into the body, which helps provide a detailed view of the blood vessels. A large area of consolidation with internal areas of breakdown and cavitation is seen in the right lower lobe with surrounding confluent nodular densities.

Chest X-ray (Figure 5) showed right lower zone consolidation and blood investigations revealed neutrophilic leukocytosis, mild liver dysfunction, urine and renal function was normal, serum homocysteine - 43.6 µmol/L (4.7 - 14.8), erythrocyte sedimentation rate (ESR) - 120 mmHr (< 15), Vitamin B12 - 192 pg/mL (239 - 931), c-reactive protein (CRP) - 16 mg/L (< 10), Folic Acid - 8 ng/mL (3 - 17), D-dimer - 2170 ng/ml (< 250) and Mantoux Test were negative. Empirical antibiotic therapy and Vitamin B12 supplementation were started within three days of admission. Chest X-ray (Figure 6) deteriorated, and the requirement for Oxygen increased. Considering clinical and radiological deterioration, CECT Thorax (Figure 7, 8) was done, which suggested - a large area of consolidation with cavitation in the right lower lobe and multiple cavitary nodules in bilateral lung fields in the mid and lower lobes.

**Figure 9:**
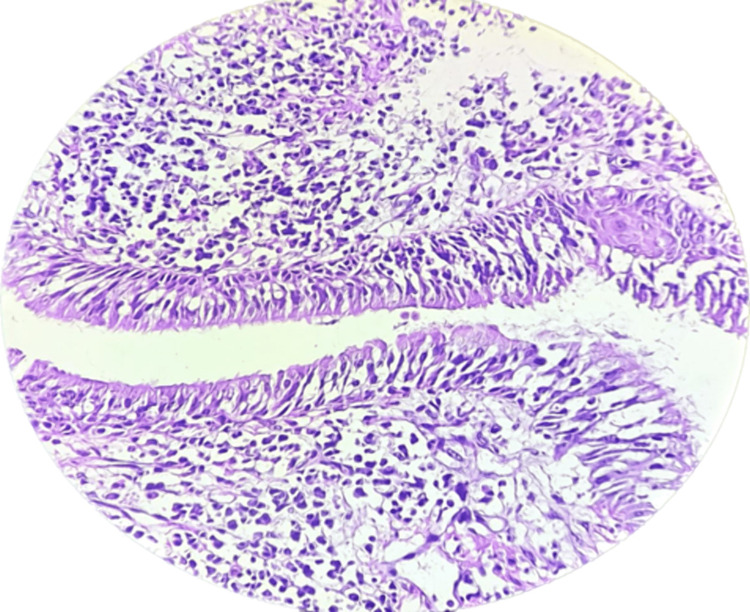
Bronchial Biopsy Biopsy - The removal of cells or tissues for examination by a pathologist. The pathologist may study the tissue under a microscope or perform other tests on the cells or tissue. Biopsy findings are suggestive of chronic inflammation.

**Figure 10:**
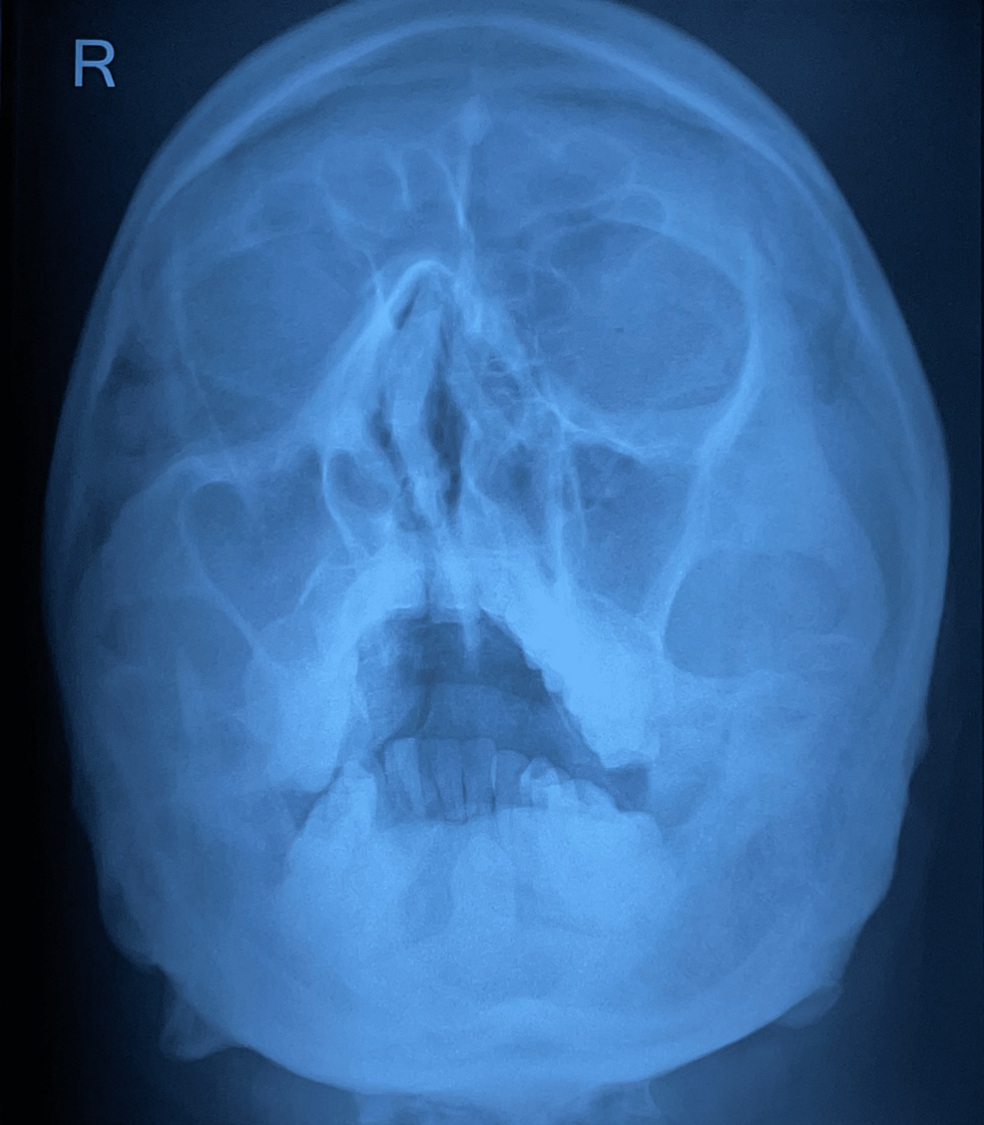
X-ray PNS X-ray - X-rays are a form of ionizing radiation, an imaging study that takes pictures of bones and soft tissues. Maxillary Sinusitis & Deviated Nasal Septum.

On day five of the presentation, our patient complained of hoarseness of voice. We suspected Pulmonary Tuberculosis, Autoimmune Disease, Systemic Vasculitis, Sarcoidosis, Bronchial Carcinoma, Lyme disease, and Nocardiosis. So, Bronchoscopy was performed, which showed severe inflammation of the mucosa, and the right vocal cord was thickened. TB PCR was negative, and a Bronchial Alveolar Lavage (BAL) cell block revealed no hemosiderin-laden macrophages and malignant cells. Bronchial biopsy suggested chronic inflammatory pathology (Figure 9). BAL Nocardia PCR, Serum angiotensin-converting enzyme (ACE) levels, and Lyme Borrelia Burgdorferi IgG were Negative. X-ray paranasal sinus (PNS) suggested Maxillary Sinusitis with Deviated Nasal Septum (Figure 10). Antinuclear antibody (ANA) and the perinuclear form of anti-neutrophil cytoplasmic antibody (P-ANCA) were also negative, while PR3 by EIAc-ANCA Serum - 200 U/mL (>=5).

**Figure 11:**
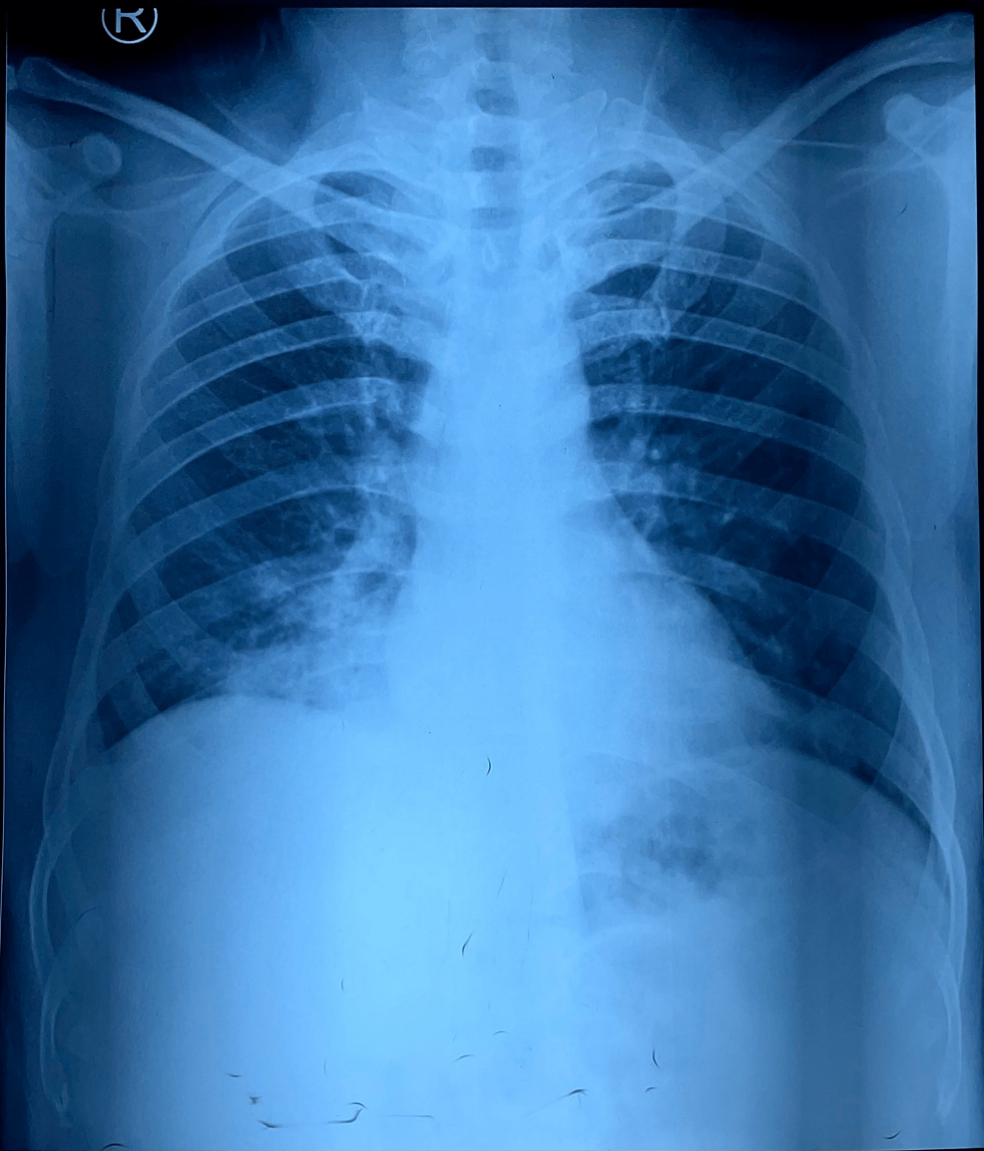
Chest X-ray on follow-up. X-ray - X-rays are a form of ionizing radiation, an imaging study that takes pictures of bones and soft tissues. Lower zone homogenous consolidation was reduced compared to previous X-rays.

Considering our patient's clinical and radiological improvement (Figure 11) from symptomatic treatment and BVAS (Birmingham Vasculitis Activity Score) of 38±3/63, he was initiated on oral Cyclophosphamide 2mg/Kg with oral steroids. The patient has been on follow-up for the last five months and will be tested for methylenetetrahydrofolate reductase (MTHFR) mutation in the next visit.

## Discussion

Published literature describes HP and CVST in GPA as having fibrosing inflammation involving the dura mater, which encases the sinuses, resulting in progressive occlusion [5]. Few studies have shown that Hyperhomocysteinemia was associated with CVST in 52% of patients and was mostly due to dietary deficiency of Vitamin B12 and methylenetetrahydrofolate reductase (MTHFR) mutation. If the link between Hyperhomocysteinemia and vitamin B12 deficiency in CVST is confirmed, it may supply a basis for preventing CVST in patients [6]. Venous involvement is not common in GPA; if patients complain of pain in their lower limbs, DVT should be ruled out [7]. Some studies provide the first true evidence of an increased risk of Venous Thromboembolism (VTE) and an increased risk of Pulmonary Embolism (PE) in patients with GPA compared to the general population [8]. In a recent study, CNS involvement was related to frequent neurological sequelae in patients of GPA [9]. The presentation of Wegener's granulomatosis can vary, and patients may initially present with isolated cranial neuritis. Physicians should be aware of this disease's atypical and uncommon presentations, which may help in early diagnosis and initiation of treatment [10].

## Conclusions

In conclusion, GPA can also uncommonly present with CNS symptoms in the form of CVST. So, in cases of LMN facial palsy, rule out systemic vasculitis along with other common causes. In cases of CVST with GPA, MTHFR mutation should be tested, as here we have reported a case of GPA with CVST caused by Hyperhomocysteinemia. In young patients, a diagnosis of GPA should be made early as it is one of the most underrecognized diseases, and early diagnosis leads to a favorable prognosis. At this point, it can only be hypothesized that Hyperhomocysteinemia and etiopathogenesis of GPA have a direct relation, as no other cases have been reported yet. Also, further research is needed to study the relationship between the same.

## Figures and Tables

**Figure 12:**
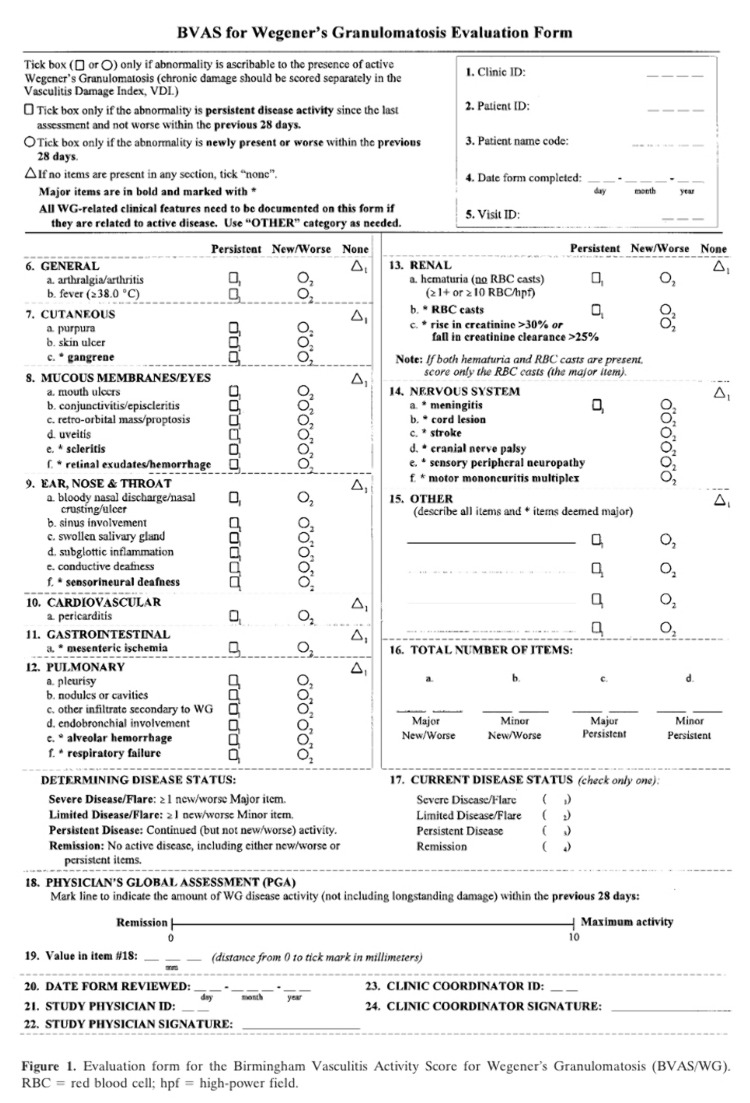
BVAS BVAS - Birmingham Vasculitis Activity Score It is designed to document new or worsening clinically active vasculitis that would be likely to require treatment, after exclusion of other causes such as infection, hypertension, etc.
